# Development and validation of a prognostic model and risk calculator for the estimation of bipolar-spectrum disorder risk in hospitalised adolescents with non-psychotic/non-bipolar mental disorders

**DOI:** 10.1038/s41380-025-03244-1

**Published:** 2025-09-29

**Authors:** Gonzalo Salazar de Pablo, Joaquim Radua, Grace Frearson, Allan H. Young, Celso Arango, Ian Kelleher, Aditya Sharma, Peter J. Uhlhaas, Marco Solmi, Paolo Fusar-Poli, Daniel Guinart, Christoph U. Correll

**Affiliations:** 1 Department of Child and Adolescent Psychiatry, Institute of Psychiatry, Psychology & Neuroscience, King’s College London, London, UK; 2 Child and Adolescent Mental Health Services, South London and Maudsley NHS Foundation Trust, London, UK; 3 Department of Child and Adolescent Psychiatry, Institute of Psychiatry and Mental Health. Hospital General Universitario Gregorio Marañón School of Medicine, Universidad Complutense, IiSGM, CIBERSAM, Madrid, Spain; 4 IMARD Group, Institut d’Investigacions Biomèdiques August Pi I Sunyer (IDIBAPS), Barcelona, Spain; 5 Biomedical Research Networking Centre Consortium on Mental Health (CIBERSAM), Instituto de Salud Carlos III, Madrid, Spain; 6 Department of Medicine, School of Medicine and Health Sciences, University of Barcelona (UB), Barcelona, Spain; 7 Centre for Affective Disorders (CfAD), Institute of Psychiatry, Psychology and Neuroscience (IoPPN), King’s College London, London, UK; 8 South London and Maudsley NHS Foundation Trust, London, UK; 9 Hospital Universitario La Paz, IdiPAZ, School of Medicine Universidad Autónoma de Madrid, CIBERSAM, Madrid, Spain; 10 Centre for Clinical Brain Sciences, University of Edinburgh, Edinburgh, UK; 11 School of Medicine, University College Dublin, Dublin, Ireland; 12 University of Oulu, Faculty of Medicine, Oulu, Finland; 13 St John of God Hospitaller Services Group, Hospitaller House, Stillorgan, Dublin, Ireland; 14 Academic Psychiatry, Translational and Clinical Research Institute, Newcastle University, Newcastle upon Tyne, UK; 15 National Specialist Adolescent Mood disorders Service (NSAMS), Cumbria, Northumberland, Tyne and Wear NHS Foundation Trust, Newcastle upon Tyne, UK; 16 Department of Child and Adolescent Psychiatry, Charité Universitätsmedizin, Berlin, Germany; 17 SCIENCES lab, Department of Psychiatry, University of Ottawa, Department of Psychiatry, Ottawa, ON Canada; 18 Regional Centre for the Treatment of Eating Disorders and On Track: The Champlain First Episode Psychosis Program, Department of Mental Health, The Ottawa Hospital, Ottawa, ON Canada; 19 Ottawa Hospital Research Institute (OHRI) Clinical Epidemiology Program University of Ottawa, Ottawa, ON Canada; 20 Early Psychosis: Interventions and Clinical-detection (EPIC) Lab, Department of Psychosis Studies, Institute of Psychiatry, Psychology & Neuroscience, King’s College London, London, UK; 21 Department of Brain and Behavioral Sciences, University of Pavia, Pavia, Italy; 22 OASIS Service, South London and Maudsley NHS Foundation Trust, London, UK; 23 National Institute for Health Research, Maudsley Biomedical Research Centre, South London and Maudsley NHS Foundation Trust, London, UK; 24 Department of Psychiatry, Hospital del Mar, Centro de Investigación Biomédica en Red de Salud Mental (CIBERSAM), Barcelona, Spain; 25 Department of Psychiatry, The Zucker Hillside Hospital, Northwell Health, Glen Oaks, NY USA; 26 Department of Psychiatry and Molecular Medicine, Zucker School of Medicine at Hofstra/ Northwell, Hempstead, NY USA; 27 Institute of Behavioral Science, The Feinstein Institutes for Medical Research, Manhasset, NY USA

**Keywords:** Bipolar disorder, Depression

## Abstract

We aimed to develop and validate a risk estimation model for developing bipolar-spectrum disorders (BSD) in psychiatrically hospitalized adolescents based on clinical characteristics, including putatively prodromal symptoms. Adolescent inpatients (ages = 12–18 years) with non-psychotic/non-BSD diagnoses were recruited for the Adolescent Mood Disorder and Psychosis Study (AMDPS), a longitudinal, prospective 5-year follow-up cohort. We assessed prevalence and severity of syndromal/subsyndromal psychopathology at baseline using the validated Bipolar Prodrome Symptom Interview and Scale–Prospective. We carried out machine learning analyses (Lasso-Cox regression analyses, LCR) to create a calculator to estimate the risk of developing BSD based on baseline demographic/comorbidity/illness/treatment characteristics. Of 105 adolescents (age = 15.6 ± 1.3 years, females = 72.4%), we observed that 18 developed BSD. The cumulated estimated risk of BSD was 5/22/29/36% at 1/2/3/4 years. BSD development was associated with presence of persistent depressive disorder (HR = 4.0, p < 0.018) at baseline, treatment with mood stabilizers (hazard ratio (HR) = 3.9, p = 0.006), and ADHD medications (HR = 3.3, p = 0.023). BSD development risk estimation calculator included the prevalence of inflated self-esteem/grandiosity (β = 0.83) and racing thoughts (β = 0.08) and the severity of overtalkativeness (β = 0.03) and increased energy (β = 0.04). For predicting BSD onset within the first 20 months, the area under the receiver operating characteristic curve (AUC) indicated acceptable to strong discrimination (cross-validation AUC = 0.72; bootstrap out-of-bag validation AUC = 0.86). Codes used in this study are provided in the R package “easy.glmnet”. In conclusion, in this prognostic model/calculator, presence and severity of subthreshold (hypo)mania-like symptoms conferred increased risk of BSD development in youth, informing preventive efforts to identify individuals at risk for BSD and improve their outcomes.

## Introduction

Bipolar disorder (BD) is a severe mental illness characterized by fluctuations in mood and energy [1]. BD is associated with significant impairments in cognitive, social, and everyday functioning [2]. BD affects >1% of the world’s population and ranks second regarding the greatest effect on days out of role functioning [3]. BD contributes to 9.9 million disability-adjusted life years (DALYS) [4]. Furthermore, individuals with BD have reduced life expectancy of up to 20 years, particularly due to suicide (accounting for 15% of deaths) and cardiovascular disease (accounting for 35–40% of deaths) [5]. However, there is a diagnostic delay of 5–10 years and significant duration of untreated illness [6], preventing prompt efforts to ameliorate poor outcomes.

Early signs and symptoms before full-blown mania have been described [7]. Such subthreshold symptoms preceding BD include disturbances in mood, circadian rhythms and behaviour [8], with a meta-analytically calculated duration of 27.1 ± 23.1 months [7]. While the presence of a clearly defined mania prodrome remains debated [9], rating scales and checklists, such as the Bipolar Prodrome Symptom Interview and Scale-Full Prospective (BPSS-FP) [10], Early Phase Inventory for Bipolar Disorders (EPIbipolar) [11], or the Semistructured Interview for Bipolar At Risk States (SIBARS) [12] have been developed to measure initial symptoms and define a bipolar risk syndrome. BD is associated with a wide range of risk factors, including family history of BD [13, 14], obstetric complications [15], adverse life events [16], anxiety and sleep problems [17]. Moreover, specific symptoms of mania and depression [10] are associated with a development of bipolar spectrum disorders (BSD, i.e., (BD-I, BD-II and BD-not otherwise specified (NOS)), ranging between 7.1 and 23.4% at 2 year follow-up [18]. According to an electronic health care data study, treatment setting is also related to risk of psychosis/BD, which by age 28 years was 1.8% for individuals without any child and adolescent mental health services (CAMHS) contact during childhood or adolescence, being 12.8% for those with prior outpatient CAMHS contact, and 24.0% for those with prior inpatient CAMHS admission [19]. Other studies have evaluated the BD prodrome retrospectively [7, 20]. However, very few studies comprehensively characterized and evaluated putative prodromal symptoms of (future) BSD, using a validated instrument [21].

The field of precision psychiatry and the number of prediction modelling studies has multiplied in recent years [22]. However, most focused on diagnostic models and other mental health conditions, including psychosis or ADHD [23]. One risk calculator developed for individuals with familial high-risk for BD found that dimensional measures of mania, depression, anxiety, and mood lability; psychosocial functioning; and parental age at mood disorder predicted the individual risk of developing BSD [24]. A second risk calculator from a 22-year birth cohort found that suicide risk, generalized anxiety disorder, parental physical abuse, and financial problems were associated with BD [25] Another study found that the best-fit model for predicting BD was a progressive sequence from nonspecific childhood antecedents, to adolescent depression, to index manic or hypomanic episode [26].

To our knowledge, no risk estimation model or calculator has been developed to evaluate the development of BSD in psychiatrically hospitalized adolescents. We aimed to prospectively evaluate the presence, severity and frequency of risk symptoms (including manic, depressive and general symptoms) plus their predictive value for future development of BSD.

Specifically, we aimed to a) evaluate the risk of adolescents initially hospitalized for the treatment of mood, anxiety and behavioral disorders but without psychotic disorder/BSD diagnoses during follow-up, and b) use machine learning techniques to develop and validate a risk estimation calculator for the development of BSD in initially hospitalized adolescents. We hypothesized that (1) a significant proportion of adolescents requiring inpatient treatment for non-psychotic/non-BSD conditions would develop BSD, and (2) presence and severity of some subthreshold mania-like symptoms would predict who has a higher risk of developing BSD.

## Methods

### Design and setting

The Adolescent Mood Disorder and Psychosis Study (AMDPS) [27] study is a longitudinal, prospective 5-year follow-up cohort study. Participants were recruited between 09/2009 and 07/2017 from the child and adolescent inpatient unit of Zucker Hillside Hospital. Zucker Hillside Hospital is a semi-urban, tertiary care facility for populations in Brooklyn and Queens in New York, as well as from more middle class and affluent regions on Long Island. After recruitment, participants were invited for follow-up interviews, typically on a yearly basis, until 01/2020. AMDPS was registered at ClinicalTrials.gov (NCT01383915). This was a STROBE-compliant [28] study (details available in eTable 1) reporting on a TRIPOD-compliant prediction model (details available in eTables 2–3) [29, 30].

The protocol, procedures, consent and assent documents were approved by the Northwell Health System Ethics Committee in accordance with the Helsinki Declaration of 1975 and UNESCO Universal Declaration on human rights. Written informed consent was obtained from all subject’s legal representatives/guardians and written assent was obtained from adolescents, obtaining informed consent if turning 18 years old during follow-up.

### Participants

Inclusion criteria for the overall AMDPS study were: (1) age 12–18 years; (2) hospitalized at the adolescent inpatient unit of Zucker Hillside Hospital, Glen Oaks, NY; (3) admission chart diagnosis of any affective or psychotic disorder, (4) subject and legal guardian (if subject<18) willing and able to provide written, informed consent/assent. Exclusion criteria were: (1) an estimated IQ < 70 as per Wide Range Achievement Test III Reading; (2) DSM-5 clinical criteria for autism-spectrum disorders or pervasive developmental disorder (chart diagnoses), and (3) history of any neurological or medical condition known to affect the brain. For the purpose of this present study, which includes adolescents with non-psychotic/non-bipolar mental disorders only, we excluded further (1) participants without complete BPSS-FP information (e.g. aborted interview); (2) participants with any DSM-5 research diagnosis of a psychotic disorder (DSM-5 295) or a non-psychotic BSD (296.40-89) (see diagnostic tools below), and (3) participants without follow-up assessments in whom BSD development could not be assessed.

### Diagnostic assessments

Psychiatric diagnoses were established via the Schedule for Affective Disorders and Schizophrenia for School-Age Children-Present and Lifetime version (K-SADS-PL) and the Structured Clinical Interview for DSM Disorders (SCID) comprising the Childhood Disorders Version of the Structured Clinical Interview for DSM Disorders KID-SCID) augmented for depressive disorders and BSD designations by the BPSS-FP (see below and eText 1 for Bipolar Spectrum Disorders characterizations), conducted separately with patients and caregivers according to DSM-5 criteria. BSD designations included BD-1 defined by the occurrence of ≥1 manic episode; BD-II defined by the occurrence of ≥1 hypomanic episode and ≥1d epressive episode, and BD-NOS defined by symptoms of BD and a change in functioning, but lacking sufficient duration or severity for a BD-I or BD-II diagnosis [31], using for BD-NOS specifically the definition from the Course and Outcome of Bipolar Youth (COBY) study, i.e., a distinct period of abnormally elevated, expansive, or irritable mood plus either ≥2 DSM-5 manic symptoms (≥3 if the mood was irritability only) that were clearly associated with the onset of abnormal mood plus a clear change in functioning; and presence of the elated and/or irritable mood and (hypo)manic symptoms for ≥4 cumulative hours and ≥4 cumulative days. Final diagnoses were determined in diagnostic consensus conferences reviewing clinical and rating scale information with the principal investigator (CUC), a board-certified child and adolescent psychiatrist with ample experience in the diagnosis of BSD. Assessments based on the patient and based on the caregiver interview were integrated counting the higher severity/frequency symptom (assuming that symptoms are more likely forgotten than invented or exaggerated). Baseline interviews were typically conducted a few days after hospitalization, while follow-up interviews were scheduled annually. To conduct AMDPS diagnostic and clinical assessments, experienced clinicians had to be certified by the study principal investigator (CUC) after having gone through a structured training program.

### Clinical assessments

The BPSS-FP [10] is a semi-structured interview that was developed to detect prodromal and subsyndromal bipolar states and to assess the evolution of BSD. Full details on the BPSS_FP are provided in eText 2. Briefly, other scales were administered to caregivers and youth including the Clinical Global Impression – Severity scale (CGI-S), which assessed the overall severity of illness, the Global Assessment of Functioning scale (GAF), which assessed the level on functionality, as well as The Montgomery-Ǻsberg Depression Rating Scale (MADRS) and the Young Mania Rating Scale (YMRS) which were used to assess affective symptoms.

### Data analysis

We used univariate Cox regression analyses (i.e., survival analysis) to assess the effects of the baseline characteristics on the risk of BSD development considering dropouts. We analyzed each variable separately.

We also carried out machine learning analyses to create a calculator that estimates the risk of development of BSD. We introduced into the model the demographic, comorbidities, illness and treatment characteristics detailed in Table 1, as well as the severity and frequency of BPSS-FP items (Table 2). We used Lasso Cox regression analyses (LCR). Relevance of a predictor within the model was defined as the absolute value of its standardized coefficient divided by the sum of the absolute values of all standardized coefficients [32].To avoid over-fitting, we used two strategies: a) a leave-one-out cross-validation, in which the cohort was iteratively divided into a training and a test sample, and b) a bootstrap out-of-bag validation, in which bootstrap samples were used for training while out-of-bag samples (i.e., individuals not selected in the bootstrap sample in that replication) were used for validation. In the training sample, we fitted and applied Gaussian/binomial lasso regressions to impute the missing continuous/binary predictors multiple times. Then, we fitted a Cox lasso regression analysis to create a prediction model (one per imputation dataset). Afterwards, in the test sample, we applied the Gaussian/binomial lasso regression analyses to input the missing predictors, and we applied the Cox lasso regressions to predict the development of BSD (averaged across imputations). At the end of the leave-one-out cross-validation, we had estimated one BSD development risk profile for each patient of the cohort, but we never used the same patients to create and validate the model. Similarly, after 1000 bootstrap replications, we had repeatedly estimated BSD development risk profile for each patient of the cohort but never used the same patients to create and validate the model. The reason for using two different strategies is that accuracy estimates from cross-validation may be less reliable than those from external dataset validation. Still, in simulations, we found that this problem might be mitigated in some scenarios when using bootstrap with out-of-bag validation (see eCode 1).

**Table 1:** Characteristics of the sample.

Variable	Sample (*n* = 105)	BSD during follow up (*n* = 18)	No BSD during follow up (*n* = 87)	HR BSD development	*P*-value
**Age** (years) mea*n* ± SD	15.4 ± 1.3	15.3 ± 1.3	15.5 ± 1.4	0.91	0.562
**Sex:** % males (N, %)	29 (27.6%)	7 (38.9%)	22 (25.3%)	0.57	0.247
**Race (N, %)**				1.08	0.711
White	54 (54.5%)	10 (55.6%)	44 (54.3%)		
Black or African American	20 (20.2%)	4 (22.2%)	16 (19.8%)		
Mixed Race	15 (15.2%)	3 (16.7%)	12 (14.8%)		
Asian or Pacific Islander	9 (9.1%)	1 (5.6%)	8 (9.9%)		
Other	1 (1.0%)	0 (0.0%)	1 (1.0%)		
**Psychopathology scores** mea*n* ± SD					
MADRS total	26.5 ± 15.1	28.8 ± 11.8	26.0 ± 15.7	1.01	0.527
CGI-S	4.2 ± 1.0	4.6 ± 1.1	4.2 ± 1.0	1.46	0.123
GAF current	28.3 ± 15.7	29.3 ± 16.2	28.1 ± 15.7	1.00	0.833
GAF high	56.6 ± 14.1	54.8 ± 16.7	56.9 ± 13.6	0.99	0.466
GAF low	23.9 ± 14.7	24.1 ± 16.5	23.8 ± 14.5	1.00	0.861
YMRS total	11.9 ± 10.5	16.1 ± 13.0	11.0 ± 9.7	1.08	**0.009**
**Psychiatric diagnoses**					
**Number of diagnoses (mean ± SD)**	3.2 ± 1.8	3.33 ± 1.9	3.14 ± 1.8	1.09	0.501
**Depressive disorders**	86 (81.9%)	14 (77.8%)	72 (82.8%)	0.74	0.601
Major depressive disorder single episode	42 (40%)	7 (38.9%)	35 (40.2%)	1.19	0.721
Major depressive disorder recurrent	20 (19.0%)	3 (16.7%)	17 (19.5%)	0.78	0.699
Major depressive disorder with psychotic features	12 (11.4%)	3 (16.7%)	9 (10.3%)	1.50	0.522
Other unspecified depressive disorder	22 (21.0%)	2 (11.1%)	20 (23.0%)	0.33	0.147
Persistent depressive disorder	12 (11.4%)	4 (22.2%)	8 (9.2%)	4.00	**0.018**
**Trauma- and stressor-related disorders**	15 (14.3%)	3 (16.7%)	12 (13.8%)	1.30	0.675
PTSD	8 (7.6%)	2 (11.1%)	6 (6.9%)	2.87	0.168
Adjustment disorder	7 (6.7%)	1 (5.6%)	6 (6.9%)	0.59	0.610
**Personality disorder traits**	19 (18.1%)	3 (16.7%)	16 (18.4%)	0.99	0.990
Borderline personality disorder traits	18 (17.1%)	3 (16.7%)	15 (17.2%)	1.03	0.963
Other personality disorder traits	3 (2.9%)	0 (0%)	3 (3.4%)	N.a.	N.a.
**Anxiety disorders**	47 (44.8%)	7 (38.9%)	40 (46.0%)	0.77	0.597
Generalized anxiety disorder	18 (17.1%)	3 (16.7%)	15 (17.2%)	1.04	0.955
Obsessive compulsive disorder and related	7 (6.7%)	2 (11.1%)	5 (5.7%)	1.38	0.688
Panic disorder	24 (22.9%)	5 (27.8%)	19 (21.8%)	1.71	0.314
Social phobia	8 (7.6%)	1 (5.6%)	7 (8.0%)	0.63	0.654
Other anxiety disorders	16 (15.2%)	0 (0%)	16 (18.4%)	0.00	0.997
**Disruptive behavior disorders**	43 (41.0%)	10 (55.6%)	33 (37.9%)	1.98	0.152
Attention-deficit/hyperactivity disorder	31 (29.5%)	8 (44.4%)	23 (26.4%)	2.35	0.075
Conduct disorder	4 (3.8%)	1 (5.6%)	3 (3.4%)	2.94	0.299
Oppositional defiant disorder	18 (17.1%)	2 (11.1%)	16 (18.4%)	0.49	0.348
Disruptive behavior disorder NOS	7 (6.7%)	3 (16.7%)	4 (4.6%)	2.95	0.090
**Substance use disorders**	13 (12.4%)	1 (5.6%)	12 (13.8%)	0.56	0.577
Alcohol use disorder	5 (4.8%)	1 (5.6%)	4 (4.6%)	1.54	0.676
Cannabis use disorder	13 (12.4%)	1 (5.6%)	12 (13.8%)	0.56	0.577
**Eating disorders**	7 (6.7%)	0 (0%)	7 (8.0%)	0.00	0.998
**Gender identity disorder**	2 (1.9%)	0 (0%)	2 (2.3%)	0.00	0.998
**Enuresis**	3 (2.9%)	1 (5.6%)	2 (2.3%)	3.46	0.231
**Attenuated Psychosis Syndrome**	27 (25.7%)	7 (38.9%)	20 (23.0%)	2.20	0.103
**Basic Symptoms (COGDIS)**	7 (6.7%)	2 (11.1%)	5 (5.7%)	1.37	0.677
**Tic disorders**	4 (3.8%)	2 (11.1%)	2 (2.3%)	3.70	0.084
**Pharmacological treatment**, (N, %)					
**Number of treatments (mean ± SD)**	2.7 ± 1.7	3.2 ± 1.7	2.6 ± 1.7	1.2	0.118
**Any treatment** (N, %)	102 (97.1%)	18 (100.0%)	84 (96.6%)	N.a	N.a.
**Antipsychotics**	65 (61.9%)	13 (72.2%)	52 (59.8%)	1.71	0.307
**Antidepressants**	63 (60.0%)	8 (44.4%)	55 (63.2%)	0.44	0.085
**Mood stabilizers**	38 (36.2%)	11 (61.1%)	27 (31.0%)	**3.88**	**0.006**
Mood stabilizers: Lithium	30 (28.6%)	8 (44.4%)	22 (25.3%)	**2.83**	**0.030**
Mood stabilizers: Other (valproate, topiramate, lamotrigine)	13 (12.4%)	4 (22.2%)	9 (10.3%)	2.23	0.162
**Anxiolytics**	24 (22.9%)	4 (22.2%)	20 (23.0%)	0.92	0.891
Anxiolytics: Benzodiazepines	21 (20.0%)	3 (16.7%)	18 (20.7%)	0.81	0.735
Anxiolytics: Antihistamines and others	6 (5.7%)	1 (5.6%)	5 (5.7%)	0.99	0.994
**ADHD medications**	13 (12.4%)	5 (27.8%)	8 (9.2%)	**3.33**	**0.023**
**Other medications**	9 (8.6%)	1 (5.6%)	8 (9.2%)	0.46	0.459

**Table 2:** Severity of BPSS-FP Mania Symptom Index, Depression Symptom Index and General Symptom Index items at baseline and association with BSD development.

BPSS-P items (M = mania; D = depression; G = general Symptom Index) mea*n* ± SD	Total (*n* = 105)	BSD during follow up (*n* = 18)	No BSD during follow up (*n* = 87)	HR BSD development	*P*-value
**M1:** Mood elevation	1.5 ± 1.7	2.6 ± 1.6	1.3 ± 1.6	**1.67**	**<0.001**
**M2:** Irritability	3.8 ± 1.4	4.4 ± 1.4	3.7 ± 1.5	1.42	0.065
**M3:** Inflated self-esteem/grandiosity	0.7 ± 1.2	1.4 ± 1.9	0.5 ± 0.9	**2.07**	**<0.001**
**M4:** Decreased need for sleep	0.7 ± 1.5	1.5 ± 2.2	0.5 ± 1.3	1.43	**<0.001**
**M5:** Overtalkativeness	1.6 ± 1.9	2.9 ± 2.2	1.3 ± 1.7	**1.57**	**<0.001**
**M6:** Racing thoughts	1.6 ± 2.0	3.3 ± 2.2	1.3 ± 1.7	**1.58**	**<0.001**
**M7:** Distractibility	3.0 ± 2.0	3.8 ± 2.1	2.9 ± 2.0	1.27	0.056
**M8:** Increased energy	0.9 ± 1.3	1.9 ± 1.6	0.7 ± 1.1	**1.60**	**<0.001**
**M9:** Increased psychomotor activity	3.8 ± 1.7	3.6 ± 1.9	3.9 ± 1.9	1.12	0.389
**M10:** Reckless or dangerous behavior	3.2 ± 1.9	4.2 ± 1.4	3.0 ± 2.0	1.19	0.133
**D1:** Depressed mood	4.8 ± 1.4	4.7 ± 1.3	4.8 ± 1.4	0.96	0.796
**D2:** Anhedonia	3.9 ± 1.8	3.9 ± 1.8	3.9 ± 1.8	1.01	0.926
**D3:** Decreased appetite	1.7 ± 2.1	1.4 ± 1.9	1.8 ± 2.1	0.89	0.348
**D4:** Increased appetite	1.7 ± 2.1	2.1 ± 2.3	1.7 ± 2.1	1.06	0.598
**D5:** Insomnia	3.1 ± 2.1	3.5 ± 2.1	3.0 ± 2.1	1.16	0.211
**D6:** Hypersomnia	1.7 ± 2.1	1.9 ± 2.1	1.7 ± 2.1	1.11	0.351
**D7**: Decreased psychomotor activity	2.0 ± 2.0	2.4 ± 1.9	2.0 ± 2.0	1.13	0.322
**D8**: Decreased energy	3.5 ± 2.0	3.9 ± 1.3	3.4 ± 2.1	1.15	0.268
**D9:** Worthlessness/guilt	3.8 ± 1.9	3.6 ± 1.9	3.9 ± 1.9	0.97	0.807
**D10:** Decreased concentration	3.2 ± 2.0	4.2 ± 1.4	3.0 ± 2.0	1.47	0.014
**D11:** Indecision	1.8 ± 1.7	2.3 ± 1.8	1.7 ± 1.7	1.2	0.146
**D12:** Suicidality	4.3 ± 2.2	3.9 ± 2.4	4.4 ± 2.2	0.88	0.224
**G1:** Mood lability	3.2 ± 2.1	4.4 ± 1.8	3.0 ± 2.1	**1.57**	**<0.001**
**G2**: Oppositionality	2.5 ± 1.9	2.9 ± 2.0	2.4 ± 1.9	1.22	0.141
**G3:** Anger/aggressiveness	3.2 ± 1.9	3.8 ± 1.8	3.1 ± 1.9	**1.38**	**0.034**
**G4:** Anxiety	3.6 ± 2.0	3.6 ± 2.1	3.7 ± 2.0	1.0	0.993
**G5:** Self-injurious behavior	2.8 ± 2.4	2.3 ± 2.6	2.9 ± 2.3	0.89	0.259
**G6**: Obsessions	1.0 ± 1.6	1.3 ± 1.6	1.0 ± 1.6	1.09	0.491
**G7:** Positive psychotic symptoms	1.4 ± 2.0	2.0 ± 2.4	1.3 ± 1.94	**1.27**	**0.021**
**G8:** Negative psychotic symptoms	0.9 ± 1.5	1.3 ± 1.8	0.8 ± 1.4	1.21	0.182
**G9:** Disorganized psychotic symptoms	0.4 ± 0.9	0.3 ± 0.7	0.4 ± 0.9	1.09	0.786

BPSS-FP: Bipolar Prodrome Symptom Interview and Scale-Full, Prospective. Severity is rated on an ordinal scale with 0 = absent, 1 = questionably present, 2 = mild, 3 = moderate, 4 = moderately severe, 5 = severe, 6 = extreme.

We then calculated the area under the curve (AUC) over time. AUC expresses the ability to separate individuals with and without a certain outcome and expresses how well the model ranks patients by BSD risk. This discriminative validity is considered ‘acceptable’ when AUC scores are between 0.7–0.8, ‘good’ between 0.8–0.9, and ‘excellent’ when >0.9 [33]. Finally, we split the sample into two groups (low-risk and high-risk) based on the estimated risk threshold associated with the lowest Akaike information criteria (AIC). Significance level was set at alpha = 0.05, and all tests were two-tailed. We provide the codes used in this study in the R package “easy.glmnet”.

## Results

### Patient flow

**Fig. 1:**
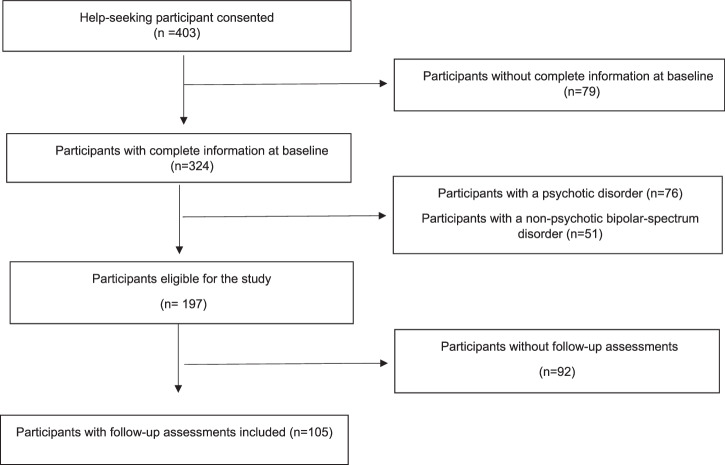
Flow diagram participants in the study. Diagram showing the number of participants who consented, exclusions due to missing data, diagnostic exclusions, and losses to follow-up. n = number of participants.

Altogether, 403 adolescents consented to participate in the AMDPS. Of these, 324 subjects had complete information at baseline. Seventy-six patients had a KID-SCID-verified psychotic disorder at baseline, and 51 had a non-psychotic BSD disorder. Of the 197 participants eligible for the study, 105 participants had follow-up information and were included in the study (Fig. 1). A comparison of sociodemographic characteristics (age, sex, race), comorbidity (depressive disorders, trauma- and stressor-related disorders, personality disorder traits, anxiety disorders, disruptive behavior disorders or substance use disorders) and pharmacological treatment (% on antipsychotics, antidepressants, mood stabilizers, anxiolytics or ADHD medications) did not reveal statistically significant or clinically meaningful differences between youths with vs. without those for whom follow- up data (all p>0.05) (comparison provided in eTable 4).

### Demographic, comorbidities, illness and treatment characteristics

The 105 adolescents with follow-up data were on average 15.4 ± 1.3 years old, 72.4% were females and 54.5% were White (Table 1). Altogether, patients had 3.2 ± 1.8 psychiatric diagnoses, including depressive disorders (81.9%), anxiety disorders (44.8%), disruptive behavior disorders (41.0%), personality disorder traits (18.1%), trauma- and stressor-related disorders (14.3%), and substance use disorders (12.4%) (Table 1). Patients were on average moderately to severely ill (CGI-S = 4.2 ± 1.0) and 97.1% received at least one psychotropic medication at baseline (mean = 2.7 ± 1.7) (antipsychotics = 61.9%, antidepressants = 60.0%, mood stabilizers = 36.2%, anxiolytics = 22.9%, and anti-ADHD medications = 12.4%) (Table 1).

### Demographic, comorbidities, illness, and treatment characteristics associated with BSD development

**Fig. 2:**
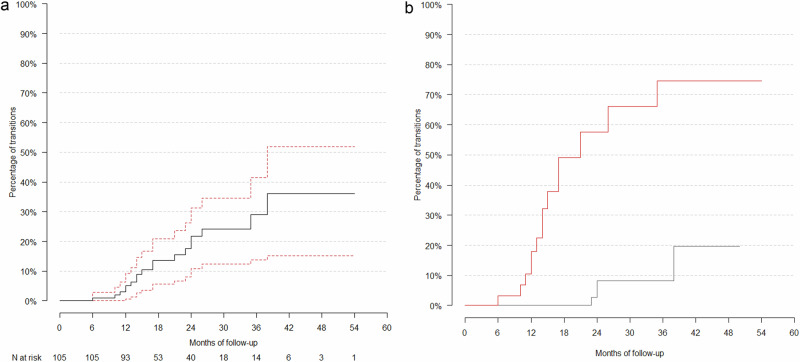
Kaplan-Meier Survival Analyses. **a** Kaplan-Meier Survival Analysis Curve of the Transition Risk to Bipolar-Spectrum Disorders. The number of patients at risk (“N at risk”) represents the number of patients still in the follow-up at each given time point (i.e., that have neither developed a BSD nor been lost to follow-up. **b** Kaplan-Meier Survival Analysis stratified by high vs. low risk groups according to the Bipolar Spectrum Disorders (BSD) risk calculator. The red line shows the percentage of transitions to BSD in patients estimated to be at high risk by the BSD calculator, while the black line shows the percentage in patients estimated to be at low risk.

Of the 105 participants with prospective data, 18 developed new-onset BSD, of which 9 developed BD-NOS, 1 BD-II, and 8 BD-I (5 without psychotic features and 3 with). As per the Kaplan-Meier estimator, which considers loss to follow-up, the cumulated estimated BSD risk was 5% at 1 year, 22% at 2 years, 29% at 3 years, and 36% at 4 years (Fig. 2).

BSD development was associated with the presence of persistent depressive disorder (HR = 4.0, p < 0.018), more baseline mood stabilizers (hazard ratio (HR) = 3.9, p = 0.006), including lithium (HR = 2.8, p = 0.03), and baseline ADHD medications (HR = 3.3, p = 0.023). BSD development was also associated with the severity of manic symptoms (HR = 1.08, p = 0.009). No associations were found with demographic characteristics (age, sex, or race), other psychiatric diagnoses, other psychopathology scores (MADRS, CGI-S), or baseline treatment with other psychopharmacologic drug classes (all p > 0.05) (see Table 2 and eTable 5 for details). See eFigure 1 for 2 Kaplan-Meier transition figures comparing the high- vs. low-risk group.

### Predictors of developing BSD, predictive value of BPSS-FP items, and risk calculator

**Fig. 3:**
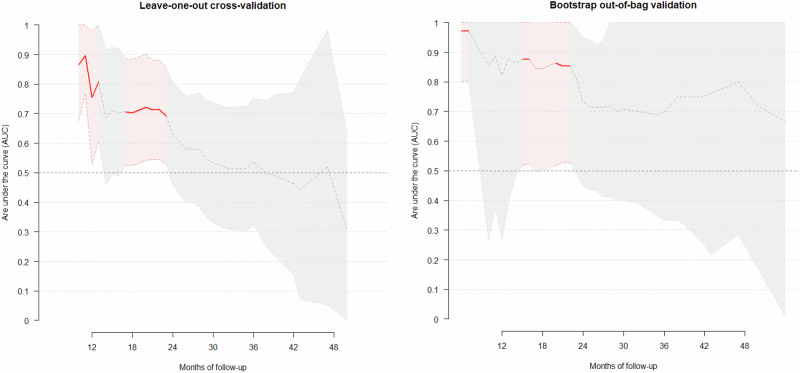
Area Under the Curve (AUC) for predictions of the Bipolar Spectrum Disorders (BSD) risk calculator over time (i.e., BSD onsets occurring since the start of the follow-up to each given month). The central dashed line represents the area under the receiver operating characteristic (AUC), and the shaded region represents its 95% confidence interval at different follow-up times. Months with an AUC statistically higher than 0.5 are represented with a thick red line. AUCs in the first months should be taken cautiously due to the small number of early developments of BSD. Months 17–18 had only borderline significance (95%CI 0.5–1) in the bootstrap out-of-bag validation and are represented with a thin red line.

According to cross-validation, BSD development risk was predicted by the prevalence of inflated self-esteem/grandiosity (M3, β = 0.83), racing thoughts (M6, β = 0.08), overtalkativeness (M5, β = 0.03), increased energy (M8, β = 0.04) and mood lability (G1, β = 0.001) (Table 3). Note the four predictors with ≥5% predicting relevance were (1) prevalence of self-esteem/grandiosity (68.1%), (2) severity of increased energy (8.2%), (3) severity of overtalkativeness (7.6%), and (4) prevalence of racing thoughts (5.3%). The AUC was statistically significant for estimating the risk of developing BSD during the first 17–23 months (Fig. 3), when 11–13 patients had developed BSD (AUC = 0.71, peaking at 0.72 in month 20, and decreasing to 0.70 in month 18 and to 0.69 in month 23, eFigure 2). Some AUCs were also statistically significant for estimating the risk during the first 13 months, although these AUCs were based on only six transitions. Bootstrap out-of-bag validation yielded the same predictors, plus a list of others (M1, M4, M10, D12, G5, MADRS, several diagnoses – note however that this validation involved creating thousands of models thus increasing the likelihood of adding other regressors). The AUC from the bootstrap out-of-bag validation was slightly higher, reaching 0.85 for estimating the risk of developing BSD during the first 20–22 months, and rising to 0.86 at month 22. Again, some AUCs were also statistically significant for estimating the risk during the first 16 months, although these AUCs were based on only nine transitions.

**Table 3:** Model: predictors selected and individual performance.

	Individual performance		Model	
	HR	95% CI	beta	Relevance
Prevalence of M3 - Inflated self-esteem/grandiosity	11.3	(4.05–31.3)	0.833	68.1%
Prevalence of M6 - Racing thoughts	6.21	(2.19–17.6)	0.079	5.3%
Severity of M5 – Overtalkativeness	1.57	(1.24–1.98)	0.030	7.6%
Severity of M8 - Increased energy	1.60	(1.25–2.04)	0.035	8.2%

The following formula underlies the online calculator available at https://www.imardgroup.com/bsd-risk-calculator, calculating where patients fall regarding the high− vs. low-risk cutoff of 0.172. The formula is 0.833 * [Prevalence of inflated self-esteem/grandiosity] + 0.079 * [Prevalence of racing thoughts] + 0.030 * [Severity of overtalkativeness] + 0.035 * [Severity of increased energy]. For descriptive purposes, we also provide the probability of developing a BSD within the first 20 months, as estimated from a logistic regression analysis (dependent variable: observed status at 20 months; independent variable: logarithm of the estimated risk plus ε = 1e-16 – we added ε because risk might be zero).

## Discussion

We observed that around 17% of adolescents developed BSD at ≤5-year follow-up, representing an estimated LCR risk of 22% at two years and 36% at four years, when taking risk factors and dropouts into account. Notably, at least in this sample, the first three years represented the highest risk period for BSD development. In univariate analyses persistent depressive disorder, and more treatment with mood stabilizers and ADHD medications (being vulnerable to cofounding by indication), as well as subthreshold mania-like symptoms were associated with BSD transition. Moreover, our BSD risk estimation model included baseline presence of inflated self-esteem/grandiosity and racing thoughts, as well as baseline severity of overtalkativeness, and increased energy. Presence/severity of these mania-like symptoms estimated BSD risk within the first 17–22 months of follow-up with an AUC of 0.71–0.85.

A previous Finnish national register study found that in adolescents aged 13–17 years old, the risk of developing BD 11–17 (mean = 5.9) years later, by age 28 (measured through electronic health care data), was 0.7% for individuals who had not attended CAMHS during childhood or adolescence, 5.0% for those with a history of any CAMHS contact, and 7.9% for those with a history of inpatient CAMHS admission [19]. Similarly, a recent study from Sweden of 114 adolescents and 114 adults from outpatient and inpatient settings reported a 7.0% development rate to BD over three years in patients diagnosed with major depressive disorder (MDD) [34]. In a longitudinal study with 3.9 years of follow-up, BP-NOS was stable in 46.1% of patients, but 38.5% transitioned to BP-I over time. Psychotic symptoms during prior depressive episodes (MDD) increased the risk of transition to BP-I 11-fold. Each individual symptom of mania increased the risk of transition to BP-I by 1.41 [35]. Notably, in that study, adolescents were significantly less likely to transition to BD in the first three years than propensity-score balanced adults (odds ratio = 0.42; 95% CI = 0.20–0.88; p = 0.02), despite having more outpatient visits at baseline. However, both groups experienced substantially reduced inpatient care following the BD diagnosis, plus a marked decline in antidepressant use without increased lithium use [34]. Although our observed transition rate to BSD was higher (17.8%) than transition rates in these two studies (7.9% in adolescents with prior inpatient care [19] and 7.0% in adolescents with MDD [34]), when restricting our analyses to BD-I and BD-II, the rate of 8.6% is comparable, although the mean follow-up of 1.5 years was shorter. However, in our study, adolescents and caregivers underwent a detailed and lengthy diagnostic interview process with validated diagnostic instruments by trained interviewers, which likely reduced the rate of underdiagnosis or late diagnosis of BD, which is more likely in clinical care [36, 37].

Our machine learning analyses, and risk estimation model successfully estimated the BSD transition risk during approximately the second year of follow-up with 0.71–0.85 accuracy. Mania-like symptoms, i.e. inflated self-esteem/grandiosity, racing thoughts, overtalkativeness, increased energy and mood lability were associated with increased risk of BSD. Symptoms of (hypo)mania, even those with lower severity, have been associated with lower self-esteem, poorer emotional states, poorer social acceptance, and lower overall quality of life [38]. Relatedly, in an adolescent inpatient sample, subthreshold mania-like symptoms also best differentiated BD depression from unipolar depression [39]. Other factors significantly associated with BD development were early age of onset of major depressive disorder (4.8 years earlier than those who without transition), and mood lability [40] among others.

Mood lability also seems to be associated with increased risk of development of BSD at follow-up. Of note, the BPSS-FP [41] screen for mania-like symptoms that map onto the BD-I and BD-II criteria that may be suprathreshold or subthreshold, but also includes mood lability as a general symptom item. The mood lability item is also included in the BPSS-Abbreviated-Prospective (BPSS-AP) [42] and in the self-rating instrument (BPSS-Abbreviated Screen (BPSS-AS) for patients and for informants [41]. Mood lability has previously been shown to predict the development of BSD in individuals with subthreshold symptoms in a risk calculator which, unlike ours, included socio-demographic factors and family history as their predictors [43], but which lacked detailed ratings of attenuated or suprathreshold symptoms of mania and depression as provided by the BPSS-FP. In offspring of individuals with BD, a history of anxiety disorders or disruptive behavior disorders has been associated with elevated risk of BSD [44]. However, while 72% of the bipolar offspring developed a lifetime DSM-IV axis I disorder and 54% a mood disorder, only 13% developed a BSD and 3% BD-1 [13].

Our risk calculator focusing on specific symptoms that confer increased BSD risk might lead to new interventions that could target preventing first hypomanic or manic episodes. Attempts to intervene at earlier stages of BD have led to initial positive outcomes [45–47] and a recent systematic review of trials for participants at high risk of developing BD found preliminary support for the efficacy of aripiprazole in reducing mood symptoms, while psychological interventions were effective for various outcomes [48]. Physical exercise has also been suggested to improve symptoms (e.g. sleep) and psychological functioning in adolescents [49]. Additional research is required before clinical recommendations can be made. It may be that some of these interventions are better suited for some at-risk groups. In any case, close monitoring is recommended for at-risk groups and, when needed, provide early interventions (e.g. for those who develop BD).

Our risk calculator has the potential to be integrated into clinical practice as a decision-support tool to enhance the early identification of adolescents at risk of developing BSD (i.e. identifying those at higher risk). Externally validating our prediction model in larger, multi-site cohorts will be essential to refine the model and ensure its robustness across different healthcare settings [23, 50]. Ultimately, this tool could contribute to precision psychiatry approaches, enabling earlier, preventive interventions that may mitigate the progression to full-threshold BSD and improve long-term outcomes in adolescents at risk [51].

Several limitations of this study should be considered. First, half of the participants lacked follow-up data. The baseline comparison of study participants with vs. without post-baseline follow-up did not show differences. However, this result does not fully exclude the possibility of differential attrition of families and patients at higher risk for BSD or those with greater clinical severity, fluctuating symptomatology, or lower engagement with follow-up assessments, which could introduce selection bias, limit generalizability, and potentially underestimate or overestimate the observed frequency of BSD development. Second, given the small sample size with few developments of BSD, power to detect statistically significant group differences for association with BSD risk may have been limited, and the results and their interpretation should be taken cautiously. Larger and longer studies following individuals at risk for BD are recommended. Nevertheless, we did find differences pointing to subsyndromal mania-like symptom presence and severity as clinical markers of future BSD risk, which is consistent with familial high-risk and database study results [52]. We also carried out machine learning analyses to estimate the risk of developing BSD from a set of baseline characteristics. Third, while the K-SADS is the gold standard to diagnose children and adolescents, some symptom collections are based on retrospective data, which may be sensitive to recall bias. However, we tried mitigating against this by interviewing caregivers as well as patients and using the higher rating of the two in case of inconsistency under the assumption that symptoms are not fabricated but rather not noticed (internalizing symptoms by caregivers, externalizing symptoms by patients), forgotten or hidden. Fourth, we included psychiatrically hospitalized adolescents. Hence, generalizability of the model to outpatients requires testing. Moreover, participants were specifically asked about symptoms rather than relying on spontaneous reporting, increasing validity and completeness of the data. Fifth, and related to this methodology, we did not include family history in our risk calculator. While family history has been described as an important predictor of BSD, the deliberate clinical risk enrichment of our sample (not previously studied) was related to psychiatric hospitalization during adolescence and not family history. Future studies including participants with both risk factors are recommended, as they may have a higher associated predictive model performance. Sixth, we did not adjust univariate group differences in the sample characteristics, which were used for descriptive purposes only, for multiple testing. Of the 105 participants with prospective data, 18 developed BSD, including 9 with BD-NOS, 1 BD-II, and 8 BD-I (5 without and 3 with psychotic features). All BSD developments occurred after hospital discharge, i.e., reported mood symptoms at baseline were not continuous with the development of BSD. Finally, other measured and unmeasured variables may have affected our results. For instance, some patients received psychotropic medications during follow-up or at the time of the assessment, which may have influenced outcomes.

In conclusion, we observed prospectively that around 17% adolescents hospitalized for psychiatric problems developed BSD at follow-up. Taking into account the baseline parameters associated with BSD development, such as prevalence of inflated self-esteem/grandiosity and racing thoughts, and severity of talkativeness, increased energy and mood lability the estimated a BSD risk was 22% at 2 years and 36% at 4 years. Our risk estimation calculator, including baseline presence and severity of mania-like symptoms (inflated self-esteem/grandiosity, racing thoughts, overtalkativeness, and increased energy) estimated risk of BSD development. These findings are hoped to inform preventive efforts with the potential to improve outcomes in adolescents at risk for and with emerging BSD. To translate these findings into clinical practice, future research should focus on piloting the risk calculator in real-world settings to evaluate its feasibility and clinical utility. Additionally, prospective external validation studies and clinical trials are needed to refine the model, address limitations, and determine its effectiveness in guiding early intervention strategies.

## Supplementary information

Supplement

## Data Availability

Data is available upon request to the corresponding author.
