# Supplementary material for: Development and validation of a prognostic model and risk calculator for the estimation of bipolar-spectrum disorder risk in hospitalised adolescents with non-psychotic/non-bipolar mental disorders
Source: Mol Psychiatry. 2025 Sep 29;31(3):1333–42. doi: 10.1038/s41380-025-03244-1 (PMC12916283; doi:10.1038/s41380-025-03244-1)
Supplement: Supplementary file 1 — Supplement [file 41380_2025_3244_MOESM1_ESM.docx]

**Supplementary material**

**-Supplementary eTable 1 –** STROBE Statement—Checklist of items in cohort studies………………………………………………………….…………………..page 2-3

**-Supplementary eTable 2 –** The TRIPOD+AI checklist…………………………….………………………………….………………………………….………………………..…page 4-5

**-Supplementary eTable 3 –** The TRIPOD+AI Abstract checklist……………………….………………………………….………………………………….………………..……page 6

**-Supplementary eTable 4 –** Comparison completers versus not completers………………………………………………………………………….……………….…...page 7

**-Supplementary eTable 5 –** Prevalence of BPSS-FP Mania Symptom Index, Depression Symptom Index and General Symptom Index items at baseline rated as moderated or higher severity and association with BSD development……………………………………………………………………….….page 8-9

**-Supplementary eFigure 1 –** Kaplan-Meier Transition Figures .……..……………………………………………………………………………………….……….….…page 10-14

**-Supplementary eFigure 2 –** Operating characteristic curves for predictions of BSD onset within the first 17-23 months ……..……….……....…page 15

**-Supplementary eText 1 -** Bipolar Spectrum Disorders characterizations……………………………………………………………………………..…...………….page 16-17

**-Supplementary eText 2 -** Bipolar Prodrome Symptom Interview and Scale-Prospective (BPSS-P) details………………………………………………….page 18

-**eCode 1**: Simulations of accuracy estimation using k-fold cross-validation, leave-one-out cross-validation, bootstrap out-of-bag validation, and new dataset validation………………………………….………………………………….………………………………….………………………………….…………………………….page 19-20

**This supplementary material has been provided by the authors to give readers additional information about their work.**

**eTable 1. STROBE Statement—Checklist of items in cohort studies ^1^**

|  | Item No | Recommendation | Page No |
| --- | --- | --- | --- |
| **Title and abstract** |  |  |  |
|  | 1 | (*a*) Indicate the study’s design with a commonly used term in the title or the abstract | 1 |
|  |  | (*b*) Provide in the abstract an informative and balanced summary of what was done and what was found | 6 |
| Introduction | | |  |
| Background/rationale | 2 | Explain the scientific background and rationale for the investigation being reported | 7 |
| Objectives | 3 | State specific objectives, including any prespecified hypotheses | 7-8 |
| Methods | | |  |
| Study design | 4 | Present key elements of study design early in the paper | 9 |
| Setting | 5 | Describe the setting, locations, and relevant dates, including periods of recruitment, exposure, follow-up, and data collection | 9 |
| Participants | 6 | (*a*) Give the eligibility criteria, and the sources and methods of selection of participants. Describe methods of follow-up | 9 |
|  |  | (*b*) For matched studies, give matching criteria and number of exposed and unexposed | 9-10 |
| Variables | 7 | Clearly define all outcomes, exposures, predictors, potential confounders, and effect modifiers. Give diagnostic criteria, if applicable | 11-12 |
| Data sources/ measurement | 8* | For each variable of interest, give sources of data and details of methods of assessment (measurement). Describe comparability of assessment methods if there is more than one group | 10-11 |
| Bias | 9 | Describe any efforts to address potential sources of bias | 10-11 |
| Study size | 10 | Explain how the study size was arrived at | 11-12 |
| Quantitative variables | 11 | Explain how quantitative variables were handled in the analyses. If applicable, describe which groupings were chosen and why | 11-12 |
| Statistical methods | 12 | (*a*) Describe all statistical methods, including those used to control for confounding | 11-12 |
|  |  | (*b*) Describe any methods used to examine subgroups and interactions | 11-12 |
|  |  | (*c*) Explain how missing data were addressed | 11-12 |
|  |  | (*d*) If applicable, explain how loss to follow-up was addressed | 11-12 |
|  |  | (*e*) Describe any sensitivity analyses | 11-12 |
| Results | | |  |
| Participants | 13* | (a) Report numbers of individuals at each stage of study—e.g.numbers potentially eligible, examined for eligibility, confirmed eligible, included in the study, completing follow-up, and analyzed | 13 |
|  |  | (b) Give reasons for non-participation at each stage | eFigure 1 |
|  |  | (c) Consider use of a flow diagram | eFigure 1 |
| Descriptive data | 14* | (a) Give characteristics of study participants (e.g.demographic, clinical, social) and information on exposures and potential confounders | 13 |
|  |  | (b) Indicate number of participants with missing data for each variable of interest | eFigure 7 |
|  |  | (c) Summarize follow-up time (e.g., average and total amount) |  |
| Outcome data | 15* | Report numbers of outcome events or summary measures over time | 13-15 |
| Main results | 16 | (*a*) Give unadjusted estimates and, if applicable, confounder-adjusted estimates and their precision (e.g., 95% confidence interval). Make clear which confounders were adjusted for and why they were included | 13-15 |
|  |  | (*b*) Report category boundaries when continuous variables were categorized | 13-15 |
|  |  | (*c*) If relevant, consider translating estimates of relative risk into absolute risk for a meaningful time period | N.a. |
| Other analyses | 17 | Report other analyses done—e.g.analyses of subgroups and interactions, and sensitivity analyses | 13-15 |
| Discussion | | |  |
| Key results | 18 | Summarize key results with reference to study objectives | 15-17 |
| Limitations | 19 | Discuss limitations of the study, taking into account sources of potential bias or imprecision. Discuss both direction and magnitude of any potential bias | 18-19 |
| Interpretation | 20 | Give a cautious overall interpretation of results considering objectives, limitations, multiplicity of analyses, results from similar studies, and other relevant evidence | 16-18 |
| Generalisability | 21 | Discuss the generalizability (external validity) of the study results | 16-18 |
| Other information | | |  |
| Funding | 22 | Give the source of funding and the role of the funders for the present study and, if applicable, for the original study on which the present article is based | 4-5 |

**eTable 2: The TRIPOD+AI checklist**

| **Section/Topic** | **Item** | **Checklist Item** | **Page** |
| --- | --- | --- | --- |
| **Title and abstract** | | | |
| Title | 1 | Identify the study as developing and/or validating a multivariable prediction model, the target population, and the outcome to be predicted. | 1 |
| Abstract | 2 | Provide a summary of objectives, study design, setting, participants, sample size, predictors, outcome, statistical analysis, results, and conclusions. | 6 |
| **Introduction** | | | |
| Background and objectives | 3a | Explain the medical context (including whether diagnostic or prognostic) and rationale for developing or validating the multivariable prediction model, including references to existing models. | 7 |
|  | 3b | Specify the objectives, including whether the study describes the development or validation of the model or both. | 7-8 |
| **Methods** | | | |
| Source of data | 4a | Describe the study design or source of data (e.g., randomized trial, cohort, or registry data), separately for the development and validation data sets, if applicable. | 9-11 |
|  | 4b | Specify the key study dates, including start of accrual; end of accrual; and, if applicable, end of follow-up. | 9-11 |
| Participants | 5a | Specify key elements of the study setting (e.g., primary care, secondary care, general population) including number and location of centres. | 9-11 |
|  | 5b | Describe eligibility criteria for participants. | 9-11 |
|  | 5c | Give details of treatments received, if relevant. | 9-11 |
| Outcome | 6a | Clearly define the outcome that is predicted by the prediction model, including how and when assessed. | 10-12 |
|  | 6b | Report any actions to blind assessment of the outcome to be predicted. | N.a. |
| Predictors | 7a | Clearly define all predictors used in developing or validating the multivariable prediction model, including how and when they were measured. | 10-12 |
|  | 7b | Report any actions to blind assessment of predictors for the outcome and other predictors. | N.a. |
| Sample size | 8 | Explain how the study size was arrived at. | 9-11 |
| Missing data | 9 | Describe how missing data were handled (e.g., complete-case analysis, single imputation, multiple imputation) with details of any imputation method. | 10-12 |
| Statistical analysis methods | 10a | Describe how predictors were handled in the analyses. | 10-12 |
|  | 10b | Specify type of model, all model-building procedures (including any predictor selection), and method for internal validation. | 10-12 |
|  | 10d | Specify all measures used to assess model performance and, if relevant, to compare multiple models. | 12 |
| Risk groups | 11 | Provide details on how risk groups were created, if done. | 12 |
| **Results** | | | |
| Participants | 13a | Describe the flow of participants through the study, including the number of participants with and without the outcome and, if applicable, a summary of the follow-up time. A diagram may be helpful. | 13 |
|  | 13b | Describe the characteristics of the participants (basic demographics, clinical features, available predictors), including the number of participants with missing data for predictors and outcome. | 13 |
| Model development | 14a | Specify the number of participants and outcome events in each analysis. | 14-15 |
|  | 14b | If done, report the unadjusted association between each candidate predictor and outcome. | N.a |
| Model specification | 15a | Present the full prediction model to allow predictions for individuals (i.e., all regression coefficients, and model intercept or baseline survival at a given time point). | 14-15 |
|  | 15b | Explain how to the use the prediction model. | 14-15 |
| Model performance | 16 | Report performance measures (with CIs) for the prediction model. | 14-15 |
| **Discussion** | | | |
| Limitations | 18 | Discuss any limitations of the study (such as nonrepresentative sample, few events per predictor, missing data). | 18 |
| Interpretation | 19b | Give an overall interpretation of the results, considering objectives, limitations, and results from similar studies, and other relevant evidence. | 16-18 |
| Implications | 20 | Discuss the potential clinical use of the model and implications for future research. | 16-18 |
| **Other information** | | | |
| Supplementary information | 21 | Provide information about the availability of supplementary resources, such as study protocol, Web calculator, and data sets. | Suppl |
| Funding | 22 | Give the source of funding and the role of the funders for the present study. | 4-5 |

**eTable 3: The TRIPOD+AI Abstract checklist**

| **ITEM** | | **ITE** | **DESCRIPTION** |  |
| --- | --- | --- | --- | --- |
| **TITLE** | | | | |
| **1** | | Identiﬁcation of the study as developing, validating, or updating a prediction model; the target population; and the outcome to be predicted. | | |
| **BACKGROUND** | | | | |
| **2** | | A brief explanation of the health care context (including whether diagnostic or prognostic) and rationale for developing, validating, orupdating the model. | | |
| **OBJECTIVES** | | | | |
| **3** | | Study objectives, including whether the study describes the development, validation, or updating of a model. For validation of an existingmodel, give the name or describe the model being validated. | | |
| **METHODS** | | | | |
| **~~4~~** | | Study design or source of data (e.g., cohort, registry, routine care data, or randomized trial), separately for the development and validationdata sets, if applicable | | |
| **5** | | Participant eligibility criteria and setting where the data were collected. | | |
| **6** | | Outcome to be predicted by the model, including time horizon of predictions in case of prognostic models (e.g., 3-year overall survival). | | |
| **7** | | Statistical model or algorithm used (e.g., logistic regression, Cox regression, random forest, or neural network) and approach for internalvalidation (for development studies) | | |
| **METHODS** | | | |  |
| **8** | | Number of participants and outcome events. | | |
| **9** | | Predictors in the ﬁnal model (for development studies) | | |
| **10** | | Performance measures, at least calibration and discrimination (with CIs), and results for added value of predictors or for model updating, if applicable. | | |
| **DISCUSSION** | | | | |
| **11** | | Overall interpretation of the results, including implications for practice or research | | |
| **REGISTRATION** | | | | |
| **12** | | Registration number and name of registry or repository. | | |

**eTable 4. Comparison of baseline demographic, illness and treatment variables in AMDPS study participants with vs without post-baseline follow-up**

| **Variable** | **Total**  **(n=197)** | **Follow-up**  **(n=105)** | **No Follow-up**  **(n=92)** | **P-value** |
| --- | --- | --- | --- | --- |
| **Age** (years) mean±SD | 15.4±1.3 | 15.4±1.3 | 15.3±1.3 | 0.808 |
| **Sex:** % males (N, %) | 51 (25.9%) | 29 (27.6%) | 22 (23.9) | 0.554 |
| **Race (N, %)** | 185 |  | 86 | 0.998 |
| White | 104 (56.1%) | 54 (54.5%) | 50 (58.1%) |  |
| Black or African American | 35 (18.9%) | 20 (20.2%) | 15 (17.4%) |  |
| Mixed Race | 27 (14.6%) | 15 (15.2%) | 12 (13.9%) |  |
| Asian or Pacific Islander | 17 (9.2%) | 9 (9.1%) | 8 (9.3%) |  |
| Other | 2 (1.1%) | 1 (1.0%) | 1 (1.1%) |  |
| **Number of diagnoses (mean ±SD)** | 3.13±1.9 | 3.2±1.8 | 3.08±2.0 | 0.810 |
| Depressive disorders | 167 (84.8%) | 86 (81.9%) | 81 (88.0%) | 0.232 |
| Trauma- and stressor-related disorders | 31 (15.7%) | 15 (14.3%) | 16 (17.4%) | 0.550 |
| Personality disorder traits | 29 (14.7%) | 19 (18.1%) | 10 (10.9%) | 0.153 |
| Anxiety disorders | 87 (44.2%) | 47 (44.8%) | 40 (43.5%) | 0.856 |
| Disruptive behaviour disorders | 71 (36.0%) | 43 (41.0%) | 28 (30.4%) | 0.125 |
| Substance use disorders | 25 (12.7%) | 13 (12.4%) | 12 (13.0%) | 0.889 |
| **Pharmacological treatment,** (N, %) |  |  |  |  |
| Antipsychotics | 121 (61.4%) | 65 (61.9%) | 56 (60.9%) | 0.882 |
| Antidepressants | 122 (61.9%) | 63 (60.0%) | 59 (64.1%) | 0.551 |
| Mood stabilizers | 69 (35.0%) | 38 (36.2%) | 31 (33.7%) | 0.714 |
| Anxiolytics | 47 (23.9%) | 24 (22.9%) | 23 (25.0%) | 0.725 |
| Anti-ADHD medications | 19 (9.6%) | 13 (12.4%) | 6 (6.5%) | 0.165 |

AMDPS: Adolescent Mood Disorder and Psychosis Study

**eTable 5. Prevalence of BPSS-FP Mania Symptom Index, Depression Symptom Index and General Symptom Index items at baseline rated as moderated or higher severity and association with BSD development**

| **BPSS-P items**  **(M=mania; D=depression; G=general Symptom Index)** mean±SD | **Total (n=105)** | **BSD during follow up (n=18)** | **No BSD during follow up (n=87)** | **HR BSD development** | **P-value** |
| --- | --- | --- | --- | --- | --- |
| **M1:** Mood elevation | 32 (30.5%) | 9 (50.0%) | 23 (26.4%) | **5.09** | **0.001** |
| **M2:** Irritability | 87 (83.7%) | 15 (88.2%) | 72 (82.8%) | 1.93 | 0.383 |
| **M3:** Inflated self-esteem/grandiosity | 10 (9.6%) | 6 (33.3%) | 4 (4.7%) | **11.26** | **<0.001** |
| **M4:** Decreased need for sleep | 14 (13.3%) | 5 (27.8%) | 9 (10.3%) | **4.79** | **0.003** |
| **M5:** Overtalkativeness | 36 (34.3%) | 11 (61.1%) | 25 (28.7%) | **4.99** | **0.001** |
| **M6:** Racing thoughts | 36 (34.3%) | 13 (72.2%) | 23 (26.4%) | **6.21** | **0.001** |
| **M7:** Distractibility | 65 (61.9%) | 14 (77.8%) | 51 (58.6%) | 2.72 | 0.078 |
| **M8:** Increased energy | 13 (12.4%) | 6 (33.3%) | 7 (8.0%) | **6.08** | **<0.001** |
| **M9:** Increased psychomotor activity | 53 (50.5%) | 11 (61.1%) | 42 (48.3%) | 1.89 | 0.188 |
| **M10:** Reckless or dangerous behavior | 24 (22.9%) | 6 (33.3%) | 18 (20.7%) | 2.32 | 0.095 |
| **Mania Symptom Index (M1-10)** | 1.6±1.0 | 2.4±1.0 | 1.5±0.9 | **2.49** | **<0.001** |
| **D1:** Depressed mood | 99 (94.3%) | 17 (94.4%) | 82 (94.3%) | 1.5 | 0.698 |
| **D2:** Anhedonia | 86 (81.9%) | 15 (83.3%) | 71 (81.6%) | 1.19 | 0.781 |
| **D3:** Decreased appetite | 33 (31.4%) | 5 (27.8%) | 28 (32.2%) | 0.8 | 0.68 |
| **D4:** Increased appetite | 35 (33.3%) | 8 (44.4%) | 27 (31.0%) | 1.6 | 0.324 |
| **D5:** Insomnia | 69 (65.7%) | 14 (77.8%) | 55 (63.2%) | 2.46 | 0.114 |
| **D6:** Hypersomnia | 37 (35.2%) | 8 (44.4%) | 29 (33.3%) | 1.64 | 0.297 |
| **D7**: Decreased psychomotor activity | 51 (48.6%) | 11 (61.1%) | 40 (46.0%) | 1.86 | 0.204 |
| **D8**: Decreased energy | 76 (72.4%) | 16 (88.9%) | 60 (69.0%) | 3.8 | 0.076 |
| **D9:** Worthlessness/guilt | 84 (80.0%) | 14 (77.8%) | 70 (80.5%) | 0.97 | 0.964 |
| **D10:** Decreased concentration | 71 (67.6%) | 15 (83.3%) | 56 (64.4%) | 2.71 | 0.115 |
| **D11:** Indecision | 38 (36.2%) | 8 (44.4%) | 30 (34.5%) | 1.92 | 0.171 |
| **D12:** Suicidality | 82 (78.1%) | 13 (72.2%) | 69 (79.3%) | 0.65 | 0.41 |
| **Depression Symptom Index (D1-12)** | 3.0±1.1 | 3.1±0.9 | 3.0±1.1 | 1.24 | 0.36 |
| **G1:** Mood lability | 69 (65.7%) | 16 (88.9%) | 53 (60.9%) | **5.20** | **0.028** |
| G2: Oppositionality | 60 (57.1%) | 12 (66.7%) | 48 (55.2%) | 1.83 | 0.228 |
| G3: Anger/aggressiveness | 73 (69.5%) | 15 (83.3%) | 58 (66.7%) | 3.1 | 0.076 |
| G4: Anxiety | 76 (72.4%) | 14 (77.8%) | 62 (71.3%) | 1.44 | 0.527 |
| G5: Self-injurious behavior | 62 (59.0%) | 8 (44.4%) | 54 (62.1%) | 0.54 | 0.197 |
| G6: Obsessions | 22 (21.0%) | 6 (33.3%) | 16 (18.4%) | 2.25 | 0.106 |
| G7: Positive psychotic symptoms | 23 (21.9%) | 7 (38.9%) | 16 (18.4%) | **2.68** | **0.039** |
| G8: Negative psychotic symptoms | 15 (14.3%) | 5 (27.8%) | 10 (11.5%) | 1.27 | 0.653 |
| G9: Disorganized psychotic symptoms | 4 (3.8%) | 1 (5.6%) | 3 (3.4%) | 1.36 | 0.626 |
| **General Symptom Index (G1-9)** | 2.1±0.9 | 2.4±0.9 | 2.02±0.9 | 1.24 | 0.36 |
| BPSS-FP: Bipolar Prodrome Symptom Interview and Scale-Full, Prospective. | | | | | |

**eFigure 1: Kaplan-Meier transition rates to Bipolar-Spectrum Disorders comparing clinical high-risk vs low-risk groups**

**A. Major Depressive Disorder: yes vs no**


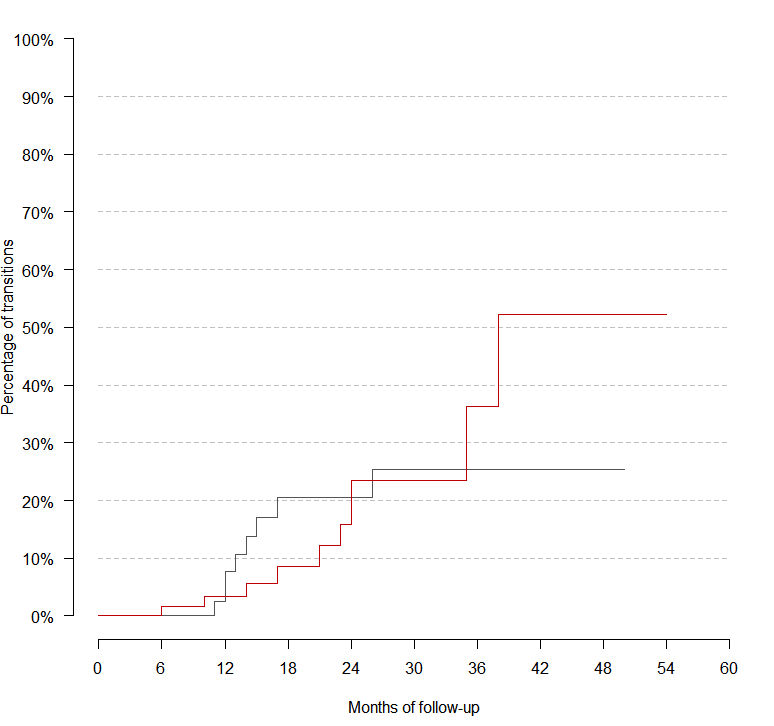


The red line shows the percentage of transitions to BSD in patients with major depressive disorder, while the black line shows the percentage in patients without.

**B.**  **Inflated self-esteem/grandiosity (M3) severity (moderate) ≥3 vs M3 severity** <**3**


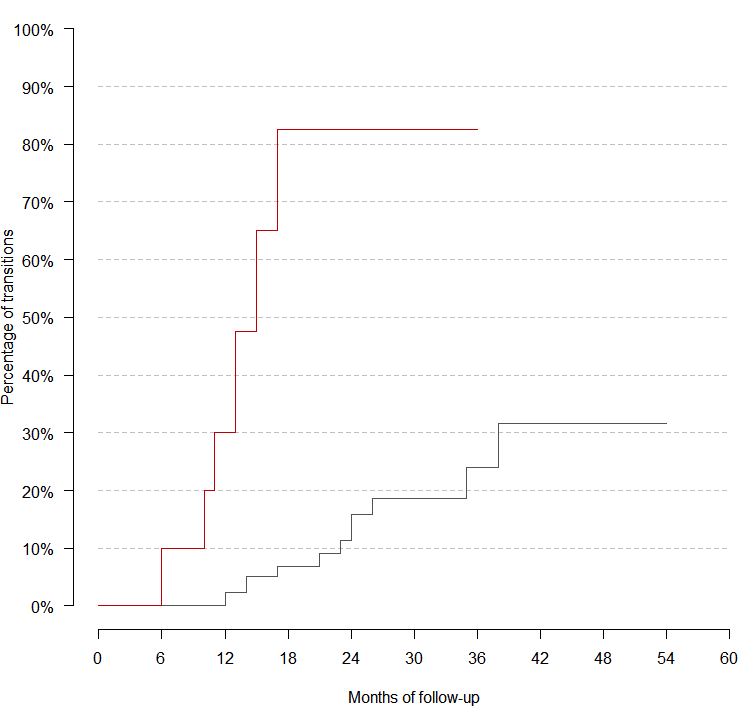


The red line shows the percentage of transitions to BSD in patients with at least moderate inflated self-esteem / grandiosity, while the black line shows the percentage in patients without.

**C. Racing thoughts (M6) severity ≥3 (moderate)** **vs M6 severity** <**3.**


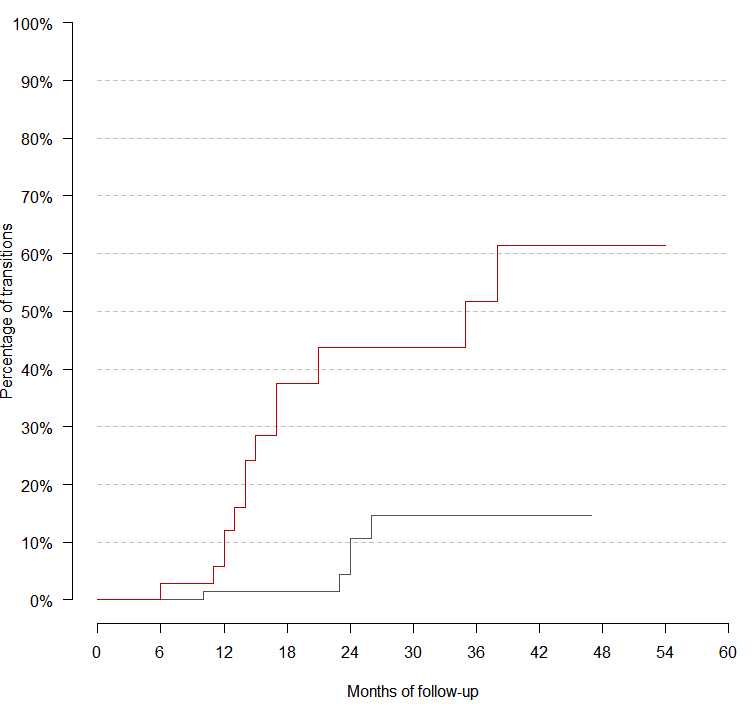


The red line shows the percentage of transitions to BSD in patients with at least moderately racing thoughts, while the black line shows the percentage in patients without.

**D. Overtalkativeness M5 severity ≥3 (moderate) vs M5 severity** <**3**


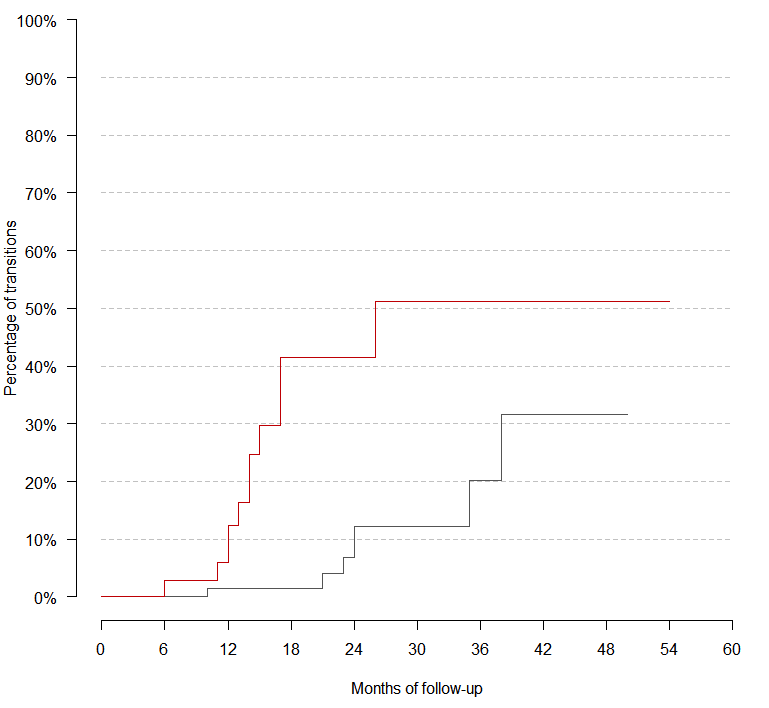


The red line shows the percentage of transitions to BSD in patients with at least moderate overtalkativeness, while the black line shows the percentage in patients without.

**E.**  **Increased energy (M8) severity ≥3 (moderate) vs M8 severity <3**


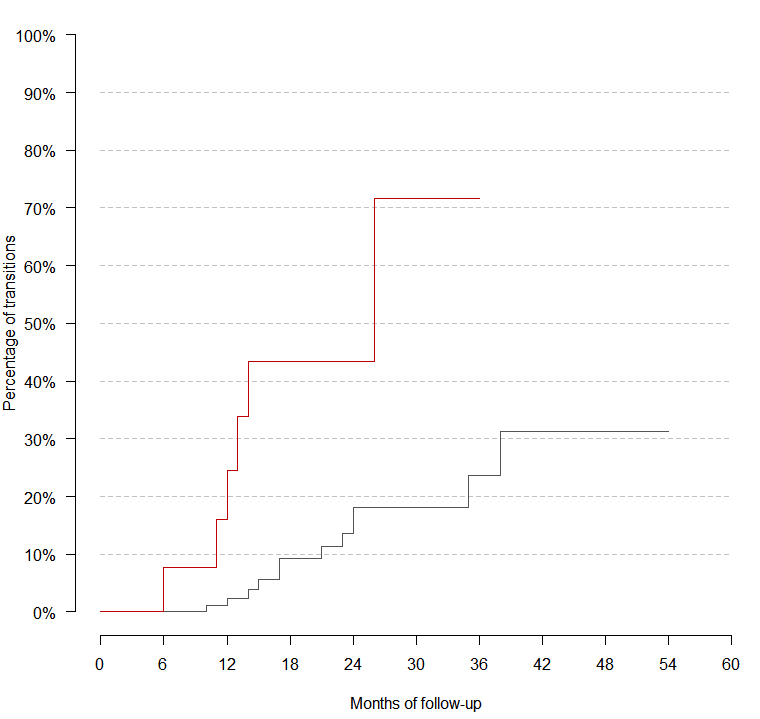


The red line shows the percentage of transitions to BSD in patients with at last moderately increased energy, while the black line shows the percentage in patients without.

**eFigure 2:** Operating characteristic curves for predictions of BSD onset within the first 17-23 months (leave-one-out cross-validation)


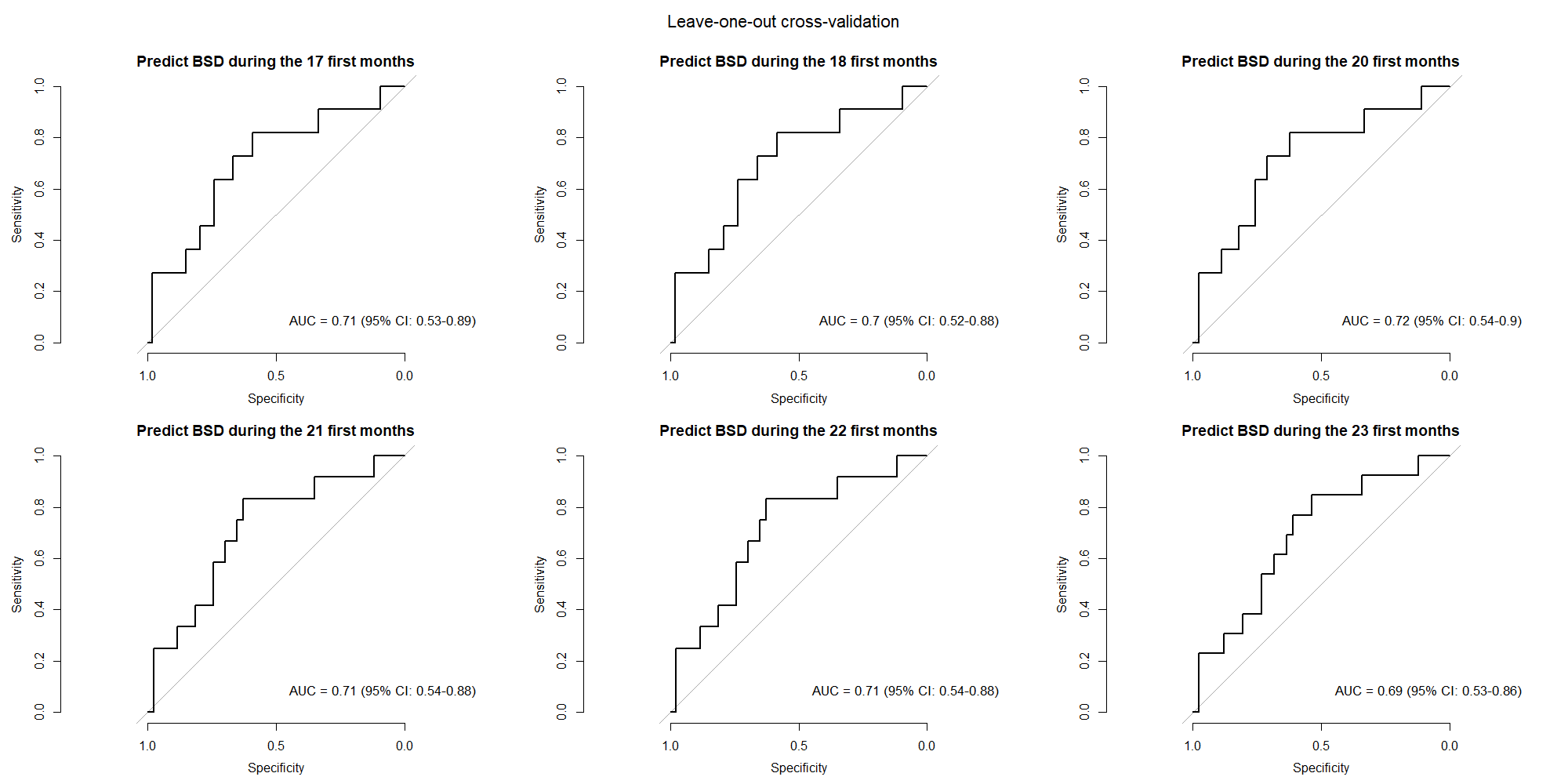


**eText 1:** Bipolar Spectrum Disorders characterization according to DSM-5^2^

Bipolar-spectrum disorders (BSD) below the level of BD-I and antecedent conditions also have a significant impact on functionality and quality of life ^3-5^. BD-I is characterized by the lifetime presence of at least one manic episode lasting at least one week; BD-II is characterized by at least one hypomanic and one major depressive episode; BD-NOS is characterized by the presence of required symptoms mania or hypomania for ≥4 hours per day, but the symptoms last ≥4 cumulative, instead of ≥4 consecutive days ^2^ (see eIntroduction for full definitions).

Early-onset BSD, i.e., onset prior to age 18, has not been studied as extensively as BD-I in adults. Notably, 60-65% of people with BD experience their first symptom onset before adulthood ^6^. However, in spite of this, the clinical presentation and the diagnosis of pediatric BSD has been controversial ^7^. Nevertheless, agreement is substantial that early-onset BD is more severe than adult-onset BD and associated with significant impairment ^8^. Moreover, childhood-onset bipolar-like symptoms seem to be a good predictor for the development of full early-onset BD and of a more severe illness course ^9, 10^.

-Bipolar I disorder (BD-I) is characterized by the occurrence of at least one manic episode, usually in association with one or more depressive episodes. One episode of mania without depression is sufficient for a diagnosis of BD-I, as long as there is no other cause of the symptoms (such as substance abuse, neurological problems, or other psychiatric disorders, like post-traumatic stress disorder) ^2^.

-BD-II is characterized by the occurrence of at least one hypomanic episode, defined as A distinct period of abnormally and persistently elevated, expansive, or irritable mood and abnormally and persistently increased activity or energy, lasting at least 4 consecutive days and present most of the day, nearly every day, and at least one depressive episode ^2^.

-BD not otherwise specified (BD-NOS), renamed “unspecified BD” in the Diagnostic and Statistical Manual of Mental Disorders, Fifth Edition (DSM-5) is characterized by symptoms that lack suﬃcient duration or severity for a BD-I or BD-II diagnosis ^2^.

This subgroup was defined in the COBY study ^11^ and included distinct periods of abnormally elevated, expansive, or irritable mood that met the following four criteria: (1) at least two DSM-IV manic symptoms in the context of elated/expansive mood (three if the mood is irritable only) that were clearly associated with the onset of abnormal mood; (2) a clear change in functioning associated with the onset of these affective symptoms; (3) the presence of elated/expansive and/or irritable mood and manic symptoms for a significant part of the day (a minimum of four hours, though not necessarily consecutive); (4) a minimum of four days (not necessarily consecutive) meeting criteria 1-3 over the subject's lifetime.

**eText 2:** Bipolar Prodrome Symptom Interview and Scale-Prospective (BPSS-P) details

The BPSS-P is comprised of a 10-item Mania Symptom Index, a 12-item Depression Symptom Index and a 6-item General Symptom Index. The BPSS-P was designed to characterize manic, depressive and general symptoms regarding the presence, severity, frequency and duration of symptoms of BD. Symptom severity is rated on an ordinal scale with 0=absent, 1=questionably present, 2=mild, 3=moderate, 4=moderately severe, 5=severe, 6=extreme. Symptom frequency is rated on an ordinal scale with 0=none, 1=<once/month, 2=once/month, 3=two-three times/month, 4=four-seven times/month, 5=eight-27 times/month, 6=>twenty-seven times/month. Symptom duration is rated on an ordinal scale with 0=absent or <1 hour, 1=one hour, 2=two-three hours, 3=four-twenty-three hours, 4=one-three days, 5=four-six days, 6=7 days or more

**eCode 1:** Simulations of accuracy estimation using k-fold cross-validation, leave-one-out cross-validation, bootstrap out-of-bag validation, and new dataset validation.

num_datasets = 5000 # Number of simulations

n = 20 # Extreme case: a tiny sample size

sim_dat = function () {

x1 = rnorm(n)

x2 = rnorm(n)

y = 2 * x1 + rnorm(n)

data.frame(x1, x2, y)

}

# Store accuracy estimates across datasets

kfoldcv_r = numeric(num_datasets)

loocv_r = numeric(num_datasets)

bootoobv_r = numeric(num_datasets)

newdatav_r = numeric(num_datasets)

# Loop over simulations

for (dataset in 1:num_datasets) {

print(dataset)

dat = sim_dat()

# K-fold cross-validation

y_pred = numeric(n)

fold = rep(1:5, n / 5)

for (b in 1:5) {

i = which(fold == b)

m = lm(y ~ x1 + x2, data = dat[-i,])

y_pred[i] = predict(m, newdata = dat[i,])

}

kfoldcv_r[dataset] = cor(dat$y, y_pred)

# Leave-one-out cross-validation

y_pred = numeric(n)

for (i in 1:n) {

m = lm(y ~ x1 + x2, data = dat[-i,])

y_pred[i] = predict(m, newdata = dat[i,])

}

loocv_r[dataset] = cor(dat$y, y_pred)

# Bootstrap out-of-bag validation

bootoobv_ri = numeric(1000)

for (b in 1:1000) {

i = sample(1:n, replace = TRUE)

m = lm(y ~ x1 + x2, data = dat[i,])

oob_y_pred = predict(m, newdata = dat[-i,])

bootoobv_ri[b] = cor(dat$y[-i], oob_y_pred)

}

bootoobv_r[dataset] = mean(na.omit(bootoobv_ri))

# New dataset validation

new_dat = sim_dat()

m = lm(y ~ x1 + x2, data = dat)

new_y_pred = predict(m, newdata = new_dat)

newdatav_r[dataset] = cor(new_dat$y, new_y_pred)

}

cat("K-fold cross-validation accuracy and 95% CI:", # 0.863 (0.649-0.948)

round(tanh(mean(atanh(kfoldcv_r))), 3),

round(quantile(kfoldcv_r, c(0.025, 0.975)), 3), "\n")

cat("Leave-one-out cross-validation accuracy and 95% CI:", # 0.866 (0.668-0.948)

round(tanh(mean(atanh(loocv_r))), 3),

round(quantile(loocv_r, c(0.025, 0.975)), 3), "\n")

cat("Bootstrap out-of-bag validation accuracy and 95% CI:", # 0.870 (0.690-0.950)

round(tanh(mean(atanh(bootoobv_r))), 3),

round(quantile(bootoobv_r, c(0.025, 0.975)), 3), "\n")

cat("New dataset validation accuracy and 95% CI:", # 0.893 (0.741-0.958)

round(tanh(mean(atanh(newdatav_r))), 3),

round(quantile(newdatav_r, c(0.025, 0.975)), 3), "\n")

**REFERENCES**

1. Benchimol EI, Smeeth L, Guttmann A, Harron K, Moher D, Petersen I *et al.* The REporting of studies Conducted using Observational Routinely-collected health Data (RECORD) statement. *PLoS Med* 2015; **12**(10)**:** e1001885.

2. American Psychiatric Association. *Diagnostic and statistical manual of mental disorders.*: Washington DC,, 2013.

3. Correll CU, Olvet DM, Auther AM, Hauser M, Kishimoto T, Carrión RE *et al.* The Bipolar Prodrome Symptom Interview and Scale-Prospective (BPSS-P): description and validation in a psychiatric sample and healthy controls. *Bipolar Disord* 2014; **16**(5)**:** 505-522.

4. Faedda GL, Marangoni C, Serra G, Salvatore P, Sani G, Vázquez GH *et al.* Precursors of bipolar disorders: a systematic literature review of prospective studies. *J Clin Psychiatry* 2015; **76**(5)**:** 614-624.

5. Salazar de Pablo G, Guinart D, Cornblatt B, Auther A, Carrión R, Carbon M *et al.* Demographic and Clinical Characteristics, Including Subsyndromal Symptoms Across Bipolar-Spectrum Disorders in Adolescents. *JCAP* 2020; **30:** 222-234.

6. Perlis RH. Misdiagnosis of bipolar disorder. *American Journal of Managed Care* 2005; **11**(SUPPL. 9)**:** S271-S274.

7. Van Meter AR, Burke C, Youngstrom EA, Faedda GL, Correll CU. The Bipolar Prodrome: Meta-Analysis of Symptom Prevalence Prior to Initial or Recurrent Mood Episodes. *J Am Acad Child Adolesc Psychiatry* 2016; **55**(7)**:** 543-555.

8. Bowie CR, Depp C, McGrath JA, Wolyniec P, Mausbach BT, Thornquist MH *et al.* Prediction of real-world functional disability in chronic mental disorders: a comparison of schizophrenia and bipolar disorder. *Am J Psychiatry* 2010; **167**(9)**:** 1116-1124.

9. Carlson GA, Pataki C. Understanding Early Age of Onset: a Review of the Last 5 Years. *Curr Psychiatry Rep* 2016; **18**(12)**:** 114.

10. Faedda GL, Baldessarini RJ, Marangoni C, Bechdolf A, Berk M, Birmaher B *et al.* An International Society of Bipolar Disorders Task force Report: Precursors and Prodromes of Bipolar Disorder. *Bipolar Disord* 2019.

11. Birmaher B, Axelson D, Strober M, Gill MK, Valeri S, Chiappetta L *et al.* Clinical course of children and adolescents with bipolar spectrum disorders. *Arch Gen Psychiatry* 2006; **63**(2)**:** 175-183.
